# Skin, Autoimmunity and Inflammation: A Comprehensive Exploration through Scientific Research

**DOI:** 10.3390/ijms242115857

**Published:** 2023-11-01

**Authors:** Federico Diotallevi, Annamaria Offidani

**Affiliations:** Dermatological Clinic, Department of Clinical and Molecular Sciences, Polytechnic Marche University, 60126 Ancona, Italy

Human skin, as the body’s largest organ, orchestrates a multifaceted interplay of cellular interactions that regulate essential physiological processes, including inflammation, immune responses, wound healing, and angiogenesis. Central to these processes is the immune system, which diligently protects the body from internal and external threats. Nevertheless, the field of dermatology comprises a complex universe of inflammatory and autoimmune skin diseases that disrupt this intricate network of cellular interactions [1].

In this Special Issue, entitled “Skin autoimmunity and inflammation”, we review skin diseases characterized by complex inflammatory mechanisms. Our mission is to examine and decipher these disorders at clinical and molecular levels to investigate present and future precise, effective, and innovative pharmacological treatments.

Psoriasis is a chronic autoimmune skin disorder that affects both adults and children. New research highlights the role of T cells, particularly Th17 and Th1, in driving the inflammatory cascade, which leads to the production of cytokines that contribute to the development of skin lesions [2]. Cytokines, such as interleukin-23 (IL-23), interleukin-17 (IL-17), and tumor necrosis factor-alpha (TNF-alpha), are key players in the disease process. Monoclonal antibodies and small molecules that inhibit these cytokines have revolutionized treatment options, offering patients safer and more effective therapies (Contribution 1).

Early treatment of the disease using these new treatments, including for the pediatric population, helps to reduce the “disease burden”, as well as the chances of psoriatic arthritis (PsA) and/or inflammatory bowel diseases that often develop as comorbidities in adulthood (Contribution 2).

Hidradenitis suppurativa (HS), like psoriasis, is also a chronic inflammatory skin condition in which autoimmune mechanisms are probably involved. However, psoriasis is commonly cleared with drugs that target the main cytokines involved (i.e., TNF-alpha, IL-17, and IL-23), yet the same drugs have reduced efficacy when treating HS [3]. This is probably because the burden of these molecules in causing the disease is less preponderant than other factors, such as bacterial colonization, particularly staphylococcus aureus, and some genetic mutations. Many other drugs with new immunological targets, such as IL-12/23, IL-17, IL-23, IL-36, C5a, CD-20, CD-40, LTA4, and CXCR1/2, are currently being studied for the treatment of HS (Contribution 3).

Microbiome manipulation and gene therapy could have potential, especially for individuals with specific condition-related genetic mutations (Contribution 4).

Vitiligo is an acquired hypopigmentation of the skin resulting from the progressive selective loss of melanocytes, and although its pathogenesis is not well defined, metabolic abnormalities, oxidative stress, inflammation, and autoimmunity are believed to contribute to its development [4]. Afamelanotide mimics α-melanocyte-stimulating hormone (MSH), which, in turn, can increase Nrf2, a gene involved in protecting the cell from oxidative stress, making it a promising treatment. Topical prostaglandins also represent an additional topical treatment option for patients with vitiligo, and it appears that their activity is greater in combination with multimodal approaches, such as skin microneedling and/or NB-UVB phototherapy. In addition, it appears that JAK-STAT inhibitors, which stimulate Sonic Hedgehog and Wnt signaling—a process involved in epidermal pigmentation and particularly in the migration, proliferation, and differentiation of melanocytes—are effective in treating the disease (Contribution 5). However, research in this field is still in the process of being developed, as is the study of systemic sclerosis (SSc).

SSc, or scleroderma, is a heterogeneous disease with variable clinical presentations. The classification of skin involvement has limited predictive value [5]. The analysis of cutaneous gene expression is promising in predicting SSc progression and responses to treatment. Molecular signatures may identify mechanisms of fibrosis and therapeutic targets. Nevertheless, caution is needed because of the heterogeneity of patients and different criteria used in the studies, and patient profiling is still far from being considered a routine procedure. Indeed, further research is needed to validate and apply gene expression profiles in clinical practice. The goal is to personalize treatments and move toward precision medicine in the management of SSc (Contribution 6).

Lichen sclerosus (LS) is a chronic inflammatory disease affecting the anogenital area, particularly in pediatric patients and postmenopausal women. Its exact cause remains unclear, posing challenges to researchers and clinicians. Proposed factors include genetics, infection, endocrine disruption, and immune system disorders [6]. As in the skin diseases mentioned above, the immunopathogenesis of LS/VLS in pediatric patients is associated with autoimmune disorders (the coexistence of autoimmune diseases and/or presence of autoantibodies against autoimmune diseases) [7]. Unfortunately, despite the availability of information on the functional role of selected molecules involved in the pathogenesis of LS/VLS, it is not easy to find meaningful data on their potential clinical utility in the diagnosis of this type of disease. Further interdisciplinary and multicenter research needs to be developed, which will allow for the identification and verification of genes, proteins, or glycoproteins that could serve as biomarkers of LS/VLS in the future (Contribution 7).

Hyper-IgE syndrome (HIES) is a category of primary immune disorders involving elevated IgE levels, eczema, and recurrent infections [8]. Genetic mutations can be identified in STAT3 (dominant) and PGM3 (recessive). Early diagnosis is complex and requires clinical and immunologic evaluations. Multi-omics analysis allows for the identification of the signaling pathways involved, enabling the development of strategies for early diagnosis, and serves as a basis for future biomarker identification (Contribution 8).

Also, a preclinical study of keloids, through a comparison of skin samples, highlighted the presence of inflammatory components. This study found that polypyrimidine tract binding (PTB) was significantly upregulated in keloid tissues. Furthermore, through selected gene silencing, PTB upregulation was demonstrated to have a central role in increasing keloid-derived fibroblast (KFb) glycolysis (Contribution 9).

Another case report presented is related to the treatment of disseminated granuloma annulare (GA). GA is a benign inflammatory disease of the skin that can be localized or disseminated. Localized GA is likely to resolve spontaneously, while disseminated GA is rare, may persist for decades, and is difficult to treat. In disseminated GA, systemic treatment may be required; all treatments used, such as cyclosporine, dapsone, hydroxychloroquine, isotretinoin, niacinamide, potassium iodide, vitamin E, or TNF-alpha blockers, have been effective in more than 50% of patients, though some may have severe side effects [9]. In the case report presented in this Special Issue focusing on the immune mechanisms underlying treatment efficacy, a good clinical response to dimethyl fumarate (DMF, Skilarence^®^ Almirall Italia S.p.A., Milan, Italy) for disseminated granuloma annulare was demonstrated (Contribution 10).

In addition, skin inflammatory reactions can also be caused by immune-related therapies, where the potential of immunotherapy meets the complexities of actual clinical cases. In this Special Issue, a clinical case of pyoderma caused by nivolumab is presented, highlighting the need for meticulous consideration when exploiting the immune system to combat non-small-cell lung cancer (Contribution 11).

All of these skin conditions often have neurological and psychiatric manifestations, as well. This Special Issue analyzes such manifestations caused by bradykinin-mediated angioedema, a condition that involves more than just the skin. This condition is characterized by increased vascular permeability due to bradykinin release. It affects various anatomical regions, including the skin and the gastrointestinal, respiratory, urinary, and genital tracts. It can be hereditary (HAE) or acquired (AAE), with known genetic mutations in some cases. HAE attacks can be triggered by trauma, surgery, menstruation, medications (e.g., ACE inhibitors), and infections. Neurological manifestations in HAE are rare and primarily documented in case reports [10]. Through the analysis of case reports and literature reviews, symptoms related to central nervous system dysfunction, peripheral nervous system dysfunction, and psychiatric disorders are analyzed in this Special Issue (Contribution 12).

Navigating the complex field of autoimmune diseases and skin inflammation, these 12 scientific articles are crucial to improving our knowledge. They challenge traditional ideas, open new avenues for treatment, and demonstrate great innovative potential in medicine. In our ongoing efforts to better understand what lies beneath the surface of the skin, we are armed with knowledge, driven by curiosity, and determined to improve the lives of those suffering from disease.
